# Western Dietary Pattern Is Associated with Irritable Bowel Syndrome in the French NutriNet Cohort

**DOI:** 10.3390/nu9090986

**Published:** 2017-09-07

**Authors:** Camille Buscail, Jean-Marc Sabate, Michel Bouchoucha, Emmanuelle Kesse-Guyot, Serge Hercberg, Robert Benamouzig, Chantal Julia

**Affiliations:** 1 Université Paris 13, Sorbonne Paris Cité, Equipe de Recherche en Epidémiologie Nutritionnelle (EREN), Centre de Recherche en Epidémiologie et Biostatistiques (CRESS), Inserm 1153, Inra U1125, Cnam, COMUE Sorbonne Paris Cité, F-93017 Bobigny, France; e.kesse@eren.smbh.univ-paris13.fr (E.K.-G.); s.hercberg@eren.smbh.univ-paris13.fr (S.H.); c.julia@eren.smbh.univ-paris13.fr (C.J.); 2 Département de Santé Publique, Hôpital Avicenne (AP-HP), F-93017 Bobigny, France; 3 Service d’ Hépato-Gastro-Entérologie, Hôpital Avicenne (AP-HP), F-93017 Bobigny, France; jean-marc.sabate@aphp.fr (J.-M.S.); michel.bouchoucha@aphp.fr (M.B.); robert.benamouzig@aphp.fr (R.B.)

**Keywords:** western diet, irritable bowel syndrome, dietary patterns, fatty food

## Abstract

**Background:** Diet appears to play a key role in the pathogenesis of the irritable bowel syndrome (IBS). Some dietary patterns (DP) could increase the risk of triggering or worsening IBS symptoms. This cross-sectional study aimed to assess the association between a posteriori derived DP and IBS in a large French population, the web-based NutriNet-Santé cohort. **Methods:** Study population included participants of the NutriNet-Santé study who completed a questionnaire based on Rome III criteria assessing IBS. A principal component analysis (PCA) was performed to identify major DPs based on 29 food groups’ consumption. Associations between DP quintiles and IBS were investigated with multivariable logistic regressions. **Results:** 44,350 participants were included, with 2423 (5.5%) presenting IBS. Three major DP were extracted using PCA, “healthy,” “western,” and “traditional.” After adjustments on confounders, the “western” DP was positively associated with IBS (OR _Q5 vs. Q1_ = 1.38, 95% CI 1.19–1.61, *p* trend < 0.0001) and the “traditional” DP was positively associated with IBS in women (OR _Q5 vs. Q1_ = 1.29 95% CI 1.08–1.54, *p* trend = 0.001). **Conclusions:** In this study, a “western” DP—highly correlated with the consumption of fatty and sugary products and snacks—was associated with a moderate increased risk of IBS.

## 1. Introduction

Irritable bowel syndrome (IBS) is one of the most frequent functional gastrointestinal disorders (FGID), defined by abdominal pain and abnormal transit conditions in the absence of detectable organic illness [1,2]. Prevalence of IBS has been estimated to be approximately 11% in the general population [3]. In France, the prevalence of IBS has been estimated at 4.7% (4.36–5.04%) [4]. Among several factors suggested to be involved in the pathogenesis of IBS, diet appears to play a key role [5,6,7,8,9]. Indeed, two thirds of IBS patients (70%) report adverse reactions to food, and 62% usually limit or exclude food items from their diet [5]. Several studies have investigated the associations between food consumption and IBS, and the food items most commonly reported by the patients as worsening or triggering IBS symptoms are the following: milk, wheat products, fatty and fried foods, caffeine, specific vegetables (cabbage, onions, peas/beans), hot spices, and alcohol [5,10,11,12,13,14,15,16]. Most studies usually study relationships between single nutrients or food components and disease, which does not allow capture of the complexity of a subject’s diet as nutrients or foods are not consumed individually but in combination in the food matrix. Comprehensive approaches involving the assessment of dietary patterns (DP) have therefore stirred considerable interest in the scientific community, as they aimed at understanding meaningful combinations of food consumption in the population [17,18]. Moreover, assessing the relationship between DP and various health outcomes in different countries appears important as several factors, including different cultures, geography and religious beliefs influence the dietary patterns of different populations [19,20]. A recent cross-sectional study performed in Iran focused on dietary patterns in relation to IBS [21], but to the best of our knowledge, no such associations have yet been studied among western populations.

The objective of the present study was to identify a posteriori DPs and to estimate their associations with IBS in a large French study, the NutriNet-Santé study.

## 2. Materials and Methods 

### 2.1. Population

Participants were selected from the NutriNet-Santé study. Briefly, the NutriNet-Santé study is a web-based prospective observational cohort, aiming at (1) assessing the relationship between nutrition and health outcomes and (2) investigating the determinants of dietary patterns and nutritional status [22]. The inclusion of, and follow-up with, volunteers aged over 18 years old are performed entirely on the internet. Inclusions started in France in May 2009 and are still ongoing with more than 158,000 participants enrolled at the time of the study. At baseline, participants completed self-administered questionnaires pertaining to socio-economic, lifestyle, health status, diet (through a set of three 24 h dietary records), physical activity, and anthropometrics data. This set of questionnaires is repeated yearly. Moreover, during follow-up, additional questionnaires are regularly proposed on various subjects pertaining to the investigation of determinants of dietary pattern or health. 

### 2.2. Ethics

The NutriNet-Santé study is set in accordance with the declaration of Helsinki and was approved by the institute Review Board of the French Institute for Health and Medical Research (00000388FWA00005831) and the Commission Nationale de l’Informatique et des Libertés (908450 and 909216). All participants provided an electronic informed consent.

### 2.3. Data Collection

#### 2.3.1. Irritable Bowel Syndrome

IBS was defined according to the Rome III criteria, through a self-administered questionnaire sent to the entire cohort between 21 June 2013 and 6 November 2013. The Rome III criteria had to be present for at least the last 6 months [23,24]. The questionnaire also contained information on the presence of organic diseases. Participants reporting any organic diseases (stomach, oesophagus or colorectal cancers, familial adenomatous polyposis coli, Crohn’s disease, coeliac disease, ulcerative colitis) or alarm symptoms (melena, hematemesis, rectal bleeding or significant unintentional weight loss in the past 3 months), were excluded from the present study.

#### 2.3.2. Dietary Data

At baseline and every year, participants were requested to complete web-based self-administered 24 h dietary records. Three non-consecutive days over a two weeks period were randomly selected for dietary records, two of them on weekdays and one on a weekend day. All participants who completed at least one set of three 24 h records between baseline and the completion of the Rome III questionnaire were eligible for the study. The more recent set of three dietary questionnaires (before the Rome III) was used to estimate dietary intakes. Each food and beverage consumed was collected according to three main meals (breakfast, lunch and dinner) and multiple possible snacking periods. Participants had to estimate the portion size for each of the items consumed using validated photographs [25]. Dietary intake was estimated using the NutriNet-Santé food composition table, including more than 3000 different foods and 70 dietary compounds reflecting foods usually consumed in the French diet [26]. This Web-based dietary assessment has been validated in several studies against traditional dietitians’ interviews and against biomarkers of nutritional status [27,28,29]. 

#### 2.3.3. Covariates

At baseline, information on age, gender, body mass index (BMI) (normal/overweight or obese), smoking status (current smoker/former smoker/nonsmoker), marital status (single/cohabiting), monthly income level (<1200 € per consumer unit (c.u.)/1200–2300 € per c.u./>2300 € per c.u.) [30] and educational level (no diploma or primary studies/secondary studies or higher educational level) were collected using self-administered questionnaires. Physical activity (PA) level was assessed using the International Physical Activity Questionnaire (IPAQ) at baseline, and Metabolic Equivalent of Task (MET) scores based on the classification of Ainsworth [31] were used to calculate a total MET for each volunteer. As proposed by the IPAQ executive committee (www.ipaq.ki.se), the minutes per week for vigorous, moderate, and walking activity were multiplied by a factor of 8, 4, and 3.3 METs, respectively. The sum of the three activity scores gives an indicator of the level of total physical activity. Additionally, participants are classified according to their total level of physical activity (1: participants highly physically active, 2: participants with intermediate level of total physical activity, 3: participants with low level of total physical activity) according to the IPAQ guidelines [31]. 

### 2.4. Statistical Analyses

A description of socio-demographical, lifestyle, anthropometrical and medical information was performed according to the IBS status (yes/no) with *t*-tests and chi-square tests, according to the type of variable. DPs were extracted using Principal Component Analysis (PCA) [32] using the 29 food groups, and the factors were rotated using orthogonal transformation (varimax rotation) [33]. The number of factors to retain in the analysis was determined using eigenvalues of each factor and Cattel’s scree test (plot of the total variance related to each pattern), as well as interpretability of the identified factors [34]. The association between food groups’ consumption and the identified factors were used to interpret the dietary patterns (DP) derived from PCA. DP were labeled based on the types of foods exhibiting the strongest correlations and having the highest factor loadings. We categorized participants by quintile of dietary pattern scores, separately in men and women (given significant interactions on gender). General characteristics of participants were compared according to quintiles of DP using the student *t*-test or Chi-square tests depending on the type of variable. To handle missing data, multiple imputations were performed [35,36]. Imputed values for physical activity (missing data = 3620, 12.5%) and income level (missing data = 2832, 9.5%) were estimated conditionally on the following variables: age, gender, marital status and educational level.

Multivariable logistic regression models were applied to estimate Odds Ratio (OR) and adjusted OR (aOR) with their 95% confidence interval (95% CI) of IBS across quintiles of DP scores, overall and according to gender. P for trend across quintiles was computed using quintiles of DP scores as an ordinal variable. Multivariable models took into account the known or suspected risk factors for IBS. Among these factors, those clearly identified in the literature were forced into the model (i.e., gender, age, educational level and physical activity), and additional factors associated with IBS with *p* < 0.20 in bivariate analyses were included. We also adjusted our models for the season of inclusion. We ran a first model minimally adjusted for baseline age (continuous) and total energy intake (Kcal, continuous). The totally adjusted model was further adjusted for educational level (no diploma or primary studies/secondary studies or higher educational level), income level (<1200 € per consumer unit (c.u.)/1200–2300 € per c.u./>2300 € per c.u.), smoking status (never smoker/former smoker/current smoker), physical activity (low, moderate, high), time between inclusion and completion (years) of IBS questionnaire, the season of inclusion, the time between dietary records and Rome III questionnaire completion and the other DPs (quintiles). A sensitivity analyses was performed by excluding participants suffering from other functional digestive disorders—i.e., Functional Constipation (FC), functional Dyspepsia (FDy) and functional diarrhoea (FD)—and assessed with the Rome III criteria within the same questionnaire. This allowed us to compare participants with IBS to controls without any FGID. All tests of significance were 2-sided and the type I error was set at 5%. All analyses were carried out using SAS software (version 9.4; SAS Institute, Inc., Cary, NC, USA) [37]. 

## 3. Results

### 3.1. Population

**Figure 1:**
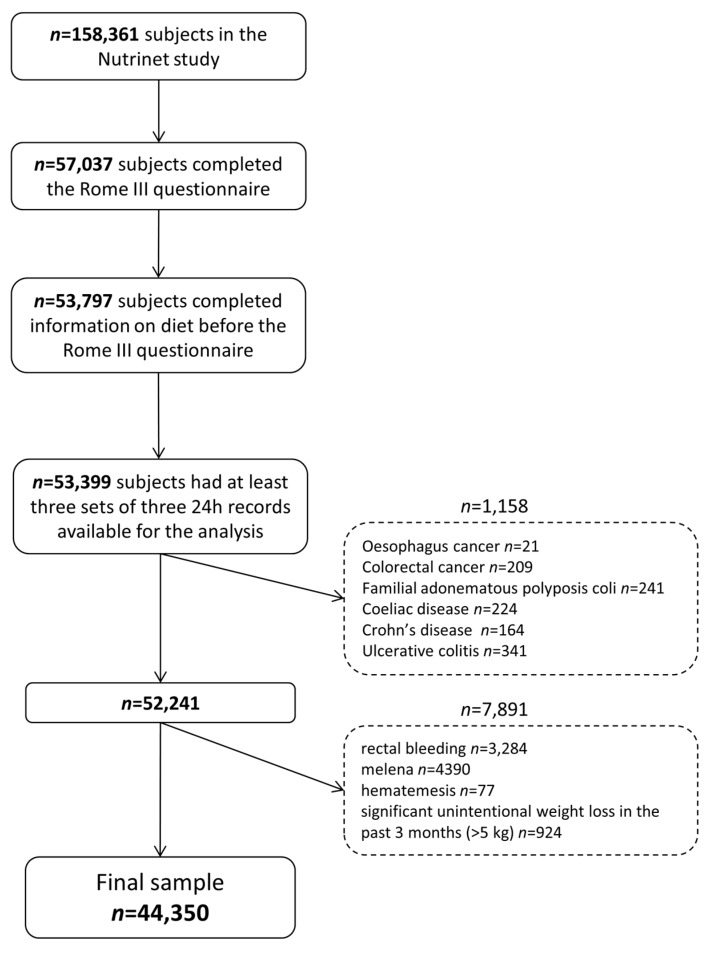
Flowchart of the study.

The final sample included 44,350 participants (Figure 1). 

Included participants were mainly women (78.3%) and the mean age was 49.7 ± 14.3 years. Overall, 2423 (5.5%) participants reported an IBS, with a higher prevalence in women compared to men (5.7% vs. 4.8%, *p* < 0.001). Compared with participants free of IBS, IBS participants were older (56.0 ± 12.0 years vs. 49.4 ± 14.3 years, with *p* < 0.0001), more often former smokers and had higher income levels (45.7% vs. 40.8%, with *p* < 0.0001) (Table 1).

### 3.2. Dietary Patterns

Description of food groups as they were used for PCA are presented in Table S1. Food groups’ loadings on the three DPs are presented in Table 2. 

Three main factors were extracted using PCA, explaining together 18.3% of the overall variance in food consumption. The first dietary pattern was termed “healthy pattern:” it was positively correlated with vegetables, whole grains, fruits, dried fruits, cereals, legumes, non-sugared beverages (water, non-sugared tea,…), and vegetable fat consumption and it was negatively correlated with meat and ham, processed meat, alcoholic beverages, milk and sweetened beverages. The second pattern was defined as a “western” pattern. It was positively correlated with fat and sugary products (including cakes, cookies and pastries), sweetened beverages and sodas, salty snacks (chips, crackers, etc.), fruit and vegetable juices, sugary cereals, starches and sauces, and negatively correlated with vegetables, dairy products, fruits, fish and seafood, poultry and eggs. The third pattern was termed “traditional” as it was consistent with a French traditional dietary pattern. It was positively correlated with bread, meat and ham, processed meat, alcoholic beverages, cheese, potatoes and tubers, animal fat, poultry, sauces, organ meat, starches and negatively correlated with sugary cereals and whole grains.

### 3.3. Characteristics of the Dietary Patterns in the Studied Population

The “healthy” profile was associated with living alone, older age, higher educational and income levels and higher PA levels, and was associated with lower proportion of current smokers and lower BMI (with all *p* values < 0.001). The “western” pattern was associated with younger age, lower BMI, lower income levels and lower PA levels but higher educational level, higher proportion of current smokers and subjects living alone (all *p* values < 0.0001). The “traditional” pattern was associated with older age, higher BMI, a greater proportion of current and former smokers, cohabiting subjects, higher incomes but lower educational level (all *p* values < 0.0001 except for educational level, *p* = 0.01) (Table 3). 

Table 4 shows results of multivariable models assessing associations between DPs and IBS. “Western” DP was overall associated with IBS (aOR_1_ = 1.14 95% CI 1.04–1.26 aOR_2_ = 1.22, 95% CI 1.09–1.37, adjusted *p* trend = 0.001) in both men (adjusted *p* trend = 0.03) and women (adjusted *p* trend = 0.02). The “traditional” DP was associated with IBS in women only (aOR_1_ = 1.08 95% CI 0.95–1.22 aOR_2_ = 1.20 95% CI 1.05–1.37, adjusted *p* trend = 0.02). No association was found between the “healthy” DP and IBS. Results were similar after removing participants who reported other functional digestive disorders (i.e., FD or FC or FDy) according to the Rome III questionnaire (*n* = 5052) (sensitivity analysis, Table S2). The “Western” pattern was significantly associated with IBS in both men and women (*p* trend < 0.01), while the “traditional” pattern was only associated with IBS in women (*p* trend < 0.001). Tables S3 and S4 show comparisons of micro and macronutrients across quintiles of the western pattern.

## 4. Discussion

In the present study, PCA was used to identify main DPs and their relationship with IBS in a large French study. Among the three major patterns identified in our study population, IBS was associated with the “western” pattern in men and women, and with the “traditional” pattern in women after adjusting on covariates. 

Both of these profiles were highly correlated with higher consumption of fatty foods (i.e., processed meat, animal fat, sauces, cheese, snacks). An association between a high-fat DP and IBS is in line with several studies which have highlighted that a large proportion of IBS patients reported their symptoms to be related to fatty food consumption [10,11,15,38]. Excessive intake of lipids as an underlying pathway in the development or worsening of IBS symptoms has been widely studied and various mechanistic hypotheses have been identified, including enhanced colonic response to lipids [39]. In normal conditions, intraluminal lipids increase perception of concurrent intestinal stimuli and modulate intestinal motor reflexes, and these effects are exaggerated in IBS patients [38,39,40]. However, available data on differences in the patterns of fat intake between IBS patients and controls are inconsistent, suggesting that the effect of diet possibly depends on its overall composition, rather than on a single component [39,41]. Moreover, “western” and “traditional” DP were both highly correlated with alcohol consumption an association with IBS that has been repeatedly highlighted [10,42,43,44].

Our “western” profile was highly correlated with consumption of fat and sugared products including cakes, cookies and pastries, desserts, sweetened beverages, sugared cereals, and with salty snacks, sauces, starches and sodas. A cross-sectional study recently performed in Iran (population of 3846 participants) has shown that a “fast food” dietary pattern was positively associated with IBS in women [21]. This dietary profile showed strong correlations with French fries, vegetable oils, meat, salt, pepper, and onions. The authors also identified a western profile, which showed no association with IBS in neither women nor men. A study performed recently in France among 380 women showed that the dietary clusters “unhealthy” and “convenience” were associated with a higher frequency of flatulence [45]. Despite some differences, the “fast-food” profile identified by Khayyatzadeh and colleagues, and the “unhealthy” cluster of Holmes and colleagues have common features with our “western” DP, especially regarding the higher amount of fats and carbohydrates intake. Moreover, they all represent a trend towards globalization of diets at the world’s level [46,47,48] (Tables S3 and S4). In France, as in other industrialized countries, shifting DP characterises a dietary transition combined with lifestyle changes. These changes, resulting in a decline in energy expenditure, have been identified as significantly associated with disability and premature deaths due to chronic non-communicable diseases which include obesity, diabetes mellitus, cardiovascular disease, hypertension and stroke, and some types of cancer [49,50]. These are partly due to higher energy intakes, increases in fat, and saturated fat intakes with higher animal source food consumption, lower intakes of fruits and vegetables, and the increase of additives and processed foods [51,52,53]. 

Unlike the “western” DP, the “traditional” profile showed an important correlation with fruits and vegetables, representing a major source of dietary fibers which are used as levers for the improvement of IBS symptoms [54]. It was also characterized by a lower proportion of pre-prepared or fast food products, which are considered risk factors for IBS [55]. These two features could partly explain why the association we found was weaker than those obtained with the “western” profile. Moreover the results were not consistent across gender for this pattern. This could partly be due to several gender and/or sex differences in IBS. There is growing evidence of the multiple points in which sex may influence the GI tract and the brain gut axis, such as mood, stress, social role, sexual hormones, visceral pain perception, motility and even genetic and immunologic microbiome [56,57,58,59]. Finally, our dietary patterns might differ from one another regarding their content in Fermentable Oligo-, Di-, Mono saccharides and Polyols (FODMAPs) or wheat. These are a particular type of carbohydrates that are poorly absorbed in the small intestine and can lead to increased luminal water retention and gas production [8,13,60,61]. FODMAPs can be found in many foods, but are more specifically present in fruits and vegetables (fructose, fructans and polyols), milk and dairy products (lactose), cereals (galactans), and prepared food (polyols) [60,62,63]. Considering this, differences in FODMAPs consumption (types and/or amounts) across DPs may partly explain our results.

The DPs we obtained in the present study are in line with those observed in previous cohorts in western countries. In the NHANES (National Health And Nutrition Examination Survey), Tseng and De Vellis identified two major DPs with PCA: the “Vegetable-fruit pattern” and the “Red meat-and-starch pattern” [64]. Fransen and colleagues in the European Prospective Investigation into Cancer and Nutrition (EPIC) cohort and Hu and colleagues in the Health Professional Follow-up Study (HPFS) identified the same two dietary profiles: “prudent pattern” and “western pattern” [65,66]. Schulze and colleagues in the EPIC cohort highlighted two profiles: ‘traditional cooking” and “fruits and vegetables.” In France, several DPs were identified across various studies in the SUpplémentation en VItamines et Minéraux Anti-oXydants (SU.VI.MAX) study, including “alcohol and meat products,” “prudent diet,” “health conscious,” “modern,” “traditional,” [32,65,66,67,68,69,70]—and in the Epidemiologic prospective cohort study of women from the MGEN national insurance plan (E3N) cohort—“Prudent,” “Western,” and “Aperifif” [71]. The “vegetable-fruit,” “health conscious,” and “prudent diet” patterns are similar to our “healthy” profile. 

Since our patterns are similar to those of other cohorts conducted among western populations, the replication of this study within other western population samples would be useful to validate our results. 

Our study had some strengths. First, to the best of our knowledge, this is the first study evaluating the association between DPs and IBS within such a large sample from the general population. Moreover, the identification of IBS was based on the Rome III criteria which was considered the gold standard at the time of inclusion [1]. The prevalence of IBS in our study is in agreement with other studies conducted among the French population and using similar identification criteria (5%) [4]. Finally, we used validated dietary collection data, using repeated and detailed dietary records [27,28,29]. 

However, some limitations need to be considered. First, the cross-sectional design of the study does not allow us to infer causality. Furthermore, the time between both exposure and outcome assessments among participants is heterogeneous. Another limitation pertains to the fact that participants were volunteers. They were probably more likely to be health conscious and have more controlled diets, and therefore may be different from a representative sample of the general population regarding dietary behaviors. For this study we selected participants with at least 3 sets of dietary questionnaires. Since IBS status was defined according to declarative data from participants, we are therefore not able to exclude that some IBS patients have an organic digestive disease. Conversely, we may have excluded some participants with IBS, due to self-reported organic disease or alarm symptoms. Nevertheless, the prevalence of IBS in our study was similar to that of the French population, which suggests a limited selection bias in relation to digestive symptoms. Moreover, we proceeded to an extensive exclusion for self-reported organic disease and alarm symptoms and given the facilitated access to medical care in France, it is unlikely that a patient with gastrointestinal symptoms for several months would not be diagnosed for an organic disease. Generalization of our results to the general population is however subject to caution. Although we controlled for several confounding factors in relation to lifestyle and digestive tract symptoms, we cannot exclude residual confounding for the interpretation of our results. Finally, aORs represent a moderate increase of the risk of IBS.

## 5. Conclusions

In this work, a “western” pattern characterized by higher consumption of fatty and sugared products, sweetened beverages, snacks and starches, was associated with an increased risk of IBS. A “traditional” French pattern was also associated with IBS in women. These results are in line with previous studies and consistent with dietary counselling in IBS. Further studies are needed to explore the nutritional content of various DPs and specifically for those suspected to play a key role in the triggering or worsening of IBS symptoms.

## Figures and Tables

**Table 1:** Characteristics of participants according to the IBS (*n* = 44,350).

Characteristics of Participants	Non Cases Participants		Participants with IBS		*p* Value *
	*n* = 41,927 (94.5%)		*n* = 2423 (5.5%)		
	*n*	%	n	%	
Gender					
Men	9183	21.9	460	19.0	<0.001
Women	32,744	78.1	1963	81.0	
Age (mean ± SD)	49.4	±14.3	56.0	±12.0	<0.0001
Educational level					
No diploma or primary school	1218	2.91	86	3.5	<0.01
Secondary	13,776	32.86	863	35.6	
High education level	26,933	64.24	1474	60.8	
BMI					
BMI < 25	29,022	70.04	1639	67.8	0.07
BMI 25–30	8949	21.60	556	23.0	
BMI ≥ 30	3465	8.36	221	9.1	
Marital status					
Single	11,146	26.58	648	26.7	0.86
Cohabiting	30,781	73.42	1775	73.3	
Smoking status					
Non smoker	21,743	51.86	1163	48.0	<0.0001
Former smoker	14,657	34.96	1000	41.3	
Current smoker	5527	13.18	260	10.7	
Monthly income level					
Less than 1200 euros per c.u.	5804	15.50	276	12.7	<0.0001
From 1200 to 2300 euros per c.u.	16,371	43.71	905	42.7	
More than 2300 euros per c.u.	15,277	40.79	992	45.7	
Physical activity level					
High	12,714	34.78	768	35.7	0.58
Moderate	15,699	42.94	922	42.8	
Low	8146	22.28	462	21.5	
Time between inclusion and IBS questionnaire (years)	3.2	±0.99	3.2	±0.97	0.12
Time between dietary records and IBS questionnaire (years)	1.0	±1.1	0.88	±1.02	<0.0001

SD, Standard Deviation; BMI, Body Mass Index; c.u, Consumer Unit. IBS, Irritable bowel syndrome; * Chi square tests or Student tests were used according to the qualitative or quantitative status of the characteristics; Missing data: Physical activity *n* = 5639 (13%); Income level *n* = 4725 (11%); Educational level *n* = 311 (<1%).

**Table 2:** Loadings of food groups in dietary pattern scores (*n* = 44,350) (see Supplementary Materials for definitions of food groups).

	DP “Healthy”	DP “Western”	DP “Traditional”
Meat, ham	−0.31		0.24
Processed meat		0.31	0.28
Fish and seafood	0.22	−0.13	
Vegetable fat	0.24	−0.15	0.27
Animal fat			0.37
Dairy products		−0.56	
Dried fruits	0.49		
Potatoes and tubers			0.41
Fruits and vegetable juices		0.25	
Starches	−0.16	0.18	
Whole grains	0.60		−0.12
Breakfast cereals	0.34		−0.14
Cakes, cookies, pastries and desserts		0.53	
Salty snacks		0.46	
Organ meat			0.16
Poultry			
Milk	−0.27		
Fruits	0.42	−0.37	0.17
Vegetables	0.46	−0.39	0.25
Sauces		0.26	0.21
Cheese		0.26	0.45
Bread	−0.34		0.62
Sugared cereals			−0.24
Confectionery	0.12		0.40
Soft non sugared beverages	0.41		0.14
Sweetened beverages and sodas	−0.11	0.35	−0.11
Alcoholic beverages		0.30	0.36
Legumes	0.34		
Eggs		−0.14	

DP, Dietary Pattern; Loading values in the range of −0.10 to 0.10 are not presented in the table.

**Table 3:** Characteristics of participants by quintiles (Q) categories of dietary pattern scores (*n* = 44,350).

Characteristics of Participants	Healthy		*p* Value *	Western		*p* Value *	Traditional		*p* Value *
	Q1 (lowest)	Q5 (highest)		Q1 (lowest)	Q5 (highest)		Q1 (lowest)	Q5 (highest)	
**Gender**									
Men	21.7	21.7		21.7	21.7		21.7	21.7	
Women	78.3	78.3		78.3	78.3		78.3	78.3	
**Age (SD)**	45.9 (14.7)	52.8 (13.1)	<0.0001	56.2 (12.4)	41.5 (13.4)	<0.0001	45.4 (14.8)	53.1 (13.1)	<0.0001
**Educational level**									
No diploma or primary school	3.7	2.5	<0.0001	4.5	1.9	<0.0001	2.6	3.2	0.02
Secondary	39.6	28.9		38.6	27.1		32.6	33.8	
High education level	57.7	68.6		57.0	71.0		64.7	63.0	
**BMI**									
BMI < 25	65.7	76.2	<0.0001	63.0	74.0	<0.0001	74.8	66.1	<0.0001
BMI 25–30	22.8	17.7		25.3	17.9		18.1	23.6	
BMI ≥ 30	11.5	6.1		11.1	8.1		7.1	10.3	
**Marital status**									
Single	26.8	29.7	<0.0001	27.3	30.0	<0.0001	34.3	22.2	<0.0001
Cohabiting	73.2	70.3		72.7	70.0		65.6	77.8	
**Smoking status**									
Non smoker	54.8	50.1	<0.0001	50.8	52.3	<0.0001	55.9	47.9	<0.0001
Former smoker	29.6	39.9		40.7	29.3		31.4	38.6	
Current smoker	15.6	10.0		8.5	18.4		12.7	13.5	
**Monthly income level**									
Less than 1200 euros per c.u.	21.3	12.1	<0.0001	13.1	18.9	<0.0001	19.3	13.2	<0.0001
From 1200 to 2300 euros per c.u.	46.6	41.4		43.7	44.4		43.4	43.2	
More than 2300 euros per c.u.	32.1	46.4		43.2	36.7		37.3	43.3	
**Physical activity**									
High	31.8	40.1	<0.0001	41.9	30.1	<0.0001	33.7	37.1	<0.0001
Moderate	41.3	43.3		39.3	43.5		43.8	41.3	
Low	26.9	16.6		18.8	26.4		22.5	21.6	

* Chi-square tests or Student tests were used according to qualitative or quantitative data; Values are mean (SD) or % as appropriate; † Quintiles are sex-specific; Chi-square tests or Student tests were used either for continuous or qualitative data; Abbreviations: BMI body mass index; c.u. consumer unit; SD standard deviation.

**Table 4:** Adjusted associations between dietary profiles and Irritable bowel syndrome (*n* = 44,350).

Dietary Profile	Model	*Number of Cases*	Q1	Q2		Q3		Q4		Q5		*p* for Trend
				OR	95% CI	OR	95% CI	OR	95% CI	OR	95% CI	
**Healthy**	**Men**	*n* *	76	89		97		108		90		
	Model 1		Ref.	1.18	0.86–1.62	1.29	0.95–1.76	1.44	1.07–1.95	1.17	0.86–1.61	0.14
	Model 2		Ref.	1.13	0.82–1.55	1.19	0.87–1.63	1.34	0.98–1.83	1.13	0.81–1.57	0.24
	**Women**	*n* *	292	385		394		416		476		
	Model 1		Ref.	1.17	1.00–1.37	1.10	0.94–1.29	1.10	0.94–1.29	1.19	1.02–1.39	0.12
	Model 2		Ref.	1.13	0.97–1.33	1.08	1.92–1.26	1.09	0.93–1.28	1.19	1.02–1.40	0.10
**Western**	**Men**	*n* *	67	89		88		107		109		
	Model 1		Ref.	1.33	0.96–1.84	1.30	0.94–1.81	1.58	1.14–2.17	1.56	1.14–2.17	<0.01
	Model 2		Ref.	1.31	0.95–1.81	1.27	0.92–1.77	1.52	1.10–2.11	1.52	1.08–2.16	0.01
	**Women**	*n* *	442	469		400		359		293		
	Model 1		Ref.	1.2	1.04–1.37	1.16	1.00–1.33	1.22	1.05–1.42	1.28	1.08–1.52	<0.01
	Model 2		Ref.	1.21	1.05–1.39	1.18	1.02–1.36	1.26	1.08–1.46	1.36	1.14–1.62	<0.001
**Traditional**	**Men**	*n* *	85	99		83		93		100		
	Model 1		Ref.	1.18	0.87–1.59	0.98	0.71–1.34	1.08	0.79–1.61	1.13	0.79–1.61	0.72
	Model 2		Ref.	1.19	0.88–1.61	1.01	0.73–1.40	1.14	0.82–1.59	1.25	0.86–1.82	0.37
	**Women**	*n* *	309	347		391		431		485		
	Model 1		Ref.	0.99	0.85–1.17	1.06	0.91–1.24	1.12	0.95–1.31	1.19	1.00–1.41	0.02
	Model 2		Ref.	1.01	0.86–1.19	1.10	0.93–1.29	1.18	1.00–1.39	1.29	1.08–1.54	<0.01

* Number of participants suffering from IBS within each sex-specific quintile of dietary profiles; † Adjusted for: age at inclusion and total energy intake; ‡ Adjusted for: model 1 + BMI, educational level, income level, smoking status, physical activity, season of inclusion, time between inclusion and completion of Rome III questionnaire and time between dietary records completion and Rome III questionnaire; Interaction on gender: 0.75 for healthy DP, <0.0001 for “western” pattern and 0.02 for “traditional” DP.

## Supplementary Materials

The following are available online at www.mdpi.com/2072-6643/9/9/986/s1, Table S1: Food grouping used in the dietary patterns (*n* = 29), Table S2: Adjusted associations between dietary profiles and irritable bowel syndrome (IBS) after the removal of other functional gastrointestinal disorders than IBS cases (*n* = 39,298), Table S3: Comparison of daily intake of macronutrients between quintiles (Q) of western pattern (*n* = 44,350), Table S4: Comparison of daily intake of micronutrients between quintiles (Q) of western pattern (*n* = 44,350).
