# Supplementary material for: Western Dietary Pattern Is Associated with Irritable Bowel Syndrome in the French NutriNet Cohort
Source: Nutrients. 2017 Sep 7;9(9):986. doi: 10.3390/nu9090986 (PMC5622746; doi:10.3390/nu9090986)
Supplement: Supplementary file 1 [file nutrients-09-00986-s001.zip › Supplemental table S1.pdf]

Supplemental Table S1. Food grouping used in the dietary patterns (n=29)

Food groups	Food items
Vegetable fat	Oils and margarine
Animal fat	Butter and other animal fat (goose or duck fat)
Sauces	Salad dressings, mayonnaise, and all types of sauces (béchamel, roquefort sauce, ...)
Cakes, cookies, pastries and desserts	Cakes, cookies, pastries, dairy desserts and ice cream
Confectionery	Candy, sweets, honey, jam, sugar, syrup, sherbet, sweetened condensed milk
Cheese	Including low fat cheese
Dairy products	Yogurts and *petits suisses* with less than 12% sugar
Milk	Any mammalian milk
Dried fruits	Dried fruits and natural oleaginous fruits
Fruits	Fruits
Fruits and vegetables juices	100% fruits or vegetables juices without added sugar
Vegetables	Any type of vegetables
Legumes	Any type of beans, lentils, lupins, peas, chickpeas, soy protein, tofu and tempeh
Potatoes and tubers	Potatoes and other tubers (cassava, sweet potato, yam, chestnut, plantain…)
Starches	Pastas, rice, semolina and flour
Bread	Bread and rusks
Sweet cereals	Sweet breakfast cereals and cereal bars
Breakfast cereals	Low sugared breakfast cereals
Whole grains	Wholemeal pasta, brown rice, wholemeal flour, wholemeal bread, wholegrain
Meat, ham	Beef, veal, lamb, mutton, pork, wild boar, rabbit, hare, horse and pork or poultry ham
Organ meat	Sausages, brains, hearts, livers, tongues, feet, sweetbreads, kidneys, viscera
Poultry	Any kind of poultry (chicken, turkey, duck, goose...)
Processed meat and fish	Terrines, pâtés, rillettes, sausage from meat or fish
Eggs	Eggs
Fish and seafood	Any kind of fish and shellfish
Salty snacks	Crackers, pretzels, chips, taco, crackers, cheese puffs, hummus, Tarama, small toasts
Alcoholic beverages	Any beverage with an alcohol content
Sweetened beverages	High-sugar fruit juices, sodas, lemonade, sweetened drinks without alcohol, syrup, ice tea, sweetened flavoured water and hot drinks
Non sugared beverages	Water (still and sparkling), light and diet drinks and unsweetened hot drinks
