# Supplementary material for: Western Dietary Pattern Is Associated with Irritable Bowel Syndrome in the French NutriNet Cohort
Source: Nutrients. 2017 Sep 7;9(9):986. doi: 10.3390/nu9090986 (PMC5622746; doi:10.3390/nu9090986)
Supplement: Supplementary file 1 [file nutrients-09-00986-s001.zip › Supplemental table S2.pdf]

Supplemental Table S2. Adjusted association between dietary profiles and irritable bowel syndrome (IBS) after the removal of other functional gastrointestinal disorders than IBS cases (n=39,298)

			Q1	Q2		Q3		Q4		Q5		
				OR	95%CI	OR	95%CI	OR	95%CI	OR	95%CI	p for trend
**Healthy**	**Men**	n*	63	76		80		93		79		
	Model 1†		Ref.	1.21	0.86-1.71	1.28	0.91-1.79	1.49	1.08-2.07	1.24	0.88-1.74	0.10
	Model 2‡		Ref.	1.15	0.82-1.62	1.18	0.84-1.66	1.39	1.00-1.94	1.19	0.83-1.70	0.20
	**Women**	n*	239	315		318		347		389		
	Model 1†		Ref.	1.17	0.99-1.40	1.09	0.92-1.30	1.13	0.96-1.35	1.19	1.00-1.41	0.13
	Model 2‡		Ref.	1.12	0.94-1.34	1.04	0.87-1.24	1.09	0.92-1.30	1.16	0.98-1.39	0.17
**Western**	**Men**	n*	53	80		70		96		92		
	Model 1†		Ref.	1.52	1.07-2.17	1.31	0.91-1.89	1.80	1.67-2.56	1.66	1.14-2.42	<0.01
	Model 2‡		Ref.	1.51	1.06-2.15	1.29	0.89-1.86	1.74	1.22-2.48	1.64	1.12-2.41	<0.01
	**Women**	n*	367	372		314		307		248		
	Model 1†		Ref.	1.13	0.97-1.31	1.07	0.92-1.26	1.22	1.04-1.44	1.23	1.02-1.47	0.02
	Model 2‡		Ref.	1.13	0.98-1.32	1.09	0.93-1.27	1.25	1.06-1.47	1.30	1.08-1.57	<0.01
**Traditional**	**Men**	n*	72	83		68		85		83		
	Model 1†		Ref.	1.16	0.84-1.60	0.93	0.66-1.32	1.16	0.82-1.63	1.08	0.73-1.58	0.74
	Model 2‡		Ref.	1.18	0.85-1.64	0.98	0.69-1.40	1.25	0.88-1.78	1.23	0.82-1.85	0.24
	**Women**	n*	248	275		315		368		402		
	Model 1†		Ref.	0.99	0.83-1.18	1.07	0.89-1.27	1.18	0.99-1.41	1.21	1.00-1.46	<0.01
	Model 2‡		Ref.	0.99	0.83-1.19	1.09	0.92-1.31	1.24	1.04-1.48	1.30	1.07-1.59	<0.001

* Number of subjects suffering from IBS within each sex-specific quintile of dietary profiles

†Adjusted for: age at inclusion, total energy intake.

‡ Adjusted for: model 1 + educational level, body mass index, income level, smoking status, physical activity, season of inclusion, time between inclusion and completion of FGID questionnaire and other DPs.
