# Supplementary material for: Western Dietary Pattern Is Associated with Irritable Bowel Syndrome in the French NutriNet Cohort
Source: Nutrients. 2017 Sep 7;9(9):986. doi: 10.3390/nu9090986 (PMC5622746; doi:10.3390/nu9090986)
Supplement: Supplementary file 1 [file nutrients-09-00986-s001.zip › Supplemental table S3.pdf]

Supplemental Table S3. Comparison of daily intake of macronutrients between quintiles (Q) of western pattern (N=44,350)

	Q1		Q2		Q3		Q4		Q5		
	mean	SD	mean	SD	mean	SD	mean	SD	mean	SD	
Total energy intake (Kcal)	1814.0	4.85	1861.9	4.76	1922.1	4.69	2027.8	4.68	2272.2	4.77	<0.0001
% energy from carbohydrates	42.8	0.08	42.0	0.08	41.5	0.08	41.0	0.08	40.3	0.08	<0.0001
% energy from lipids	35.3	0.07	37.5	0.07	38.4	0.07	39.1	0.07	39.9	0.07	<0.0001
% energy from proteins	19.6	0.04	17.5	0.04	16.6	0.04	15.8	0.04	14.9	0.04	<0.0001
MUFA	28.0	0.08	29.0	0.08	29.2	0.08	29.4	0.08	29.4	0.08	<0.0001
PUFA	11.2	0.05	11.1	0.05	11.0	0.04	10.8	0.05	10.6	0.05	<0.0001
SFA	28.5	0.09	31.2	0.09	32.4	0.08	33.4	0.09	34.7	0.09	<0.0001
Cholesterol	306.3	1.40	304.2	1.37	308.9	1.35	308.5	1.37	315.4	1.49	<0.0001
Omega 3	1.6	0.01	1.4	0.01	1.3	0.01	1.3	0.01	1.2	0.01	<0.0001
Omega 6	9.0	0.04	9.0	0.04	9.0	0.04	8.9	0.04	8.8	0.05	0.02
Animal proteins	60.9	0.19	54.4	0.18	51.9	0.18	50.0	0.18	48.2	0.20	<0.0001
Vegetal proteins	26.2	0.07	25.4	0.07	24.9	0.07	24.2	0.07	22.5	0.08	<0.0001
Complex carbohydrates	104.7	0.30	107.0	0.30	106.1	0.30	103.7	0.30	98.6	0.32	<0.0001
Simple carbohydrates	92.4	0.29	86.8	0.29	85.0	0.29	85.4	0.29	86.5	0.31	<0.0001
Fibers	22.8	0.07	20.2	0.07	18.9	0.06	17.8	0.07	15.7	0.07	<0.0001
% energy from alcohol	1.9	0.04	2.7	0.04	3.3	0.04	3.9	0.04	4.7	0.04	<0.0001
Water	2351.6	6.67	2107.9	6.54	2020.6	6.47	1962.4	6.53	1867.8	7.12	<0.0001

ANCOVA tests controlled for gender, age, total energy intake except for total energy intake, lipids, proteins and carbohydrates

Abbreviations: MUFA Mono-unsaturated fatty acid; PUFA Poly-unsaturated Fatty Acid; SFA Saturated Fatty Acid; SD standard deviation
