# Supplementary material for: Western Dietary Pattern Is Associated with Irritable Bowel Syndrome in the French NutriNet Cohort
Source: Nutrients. 2017 Sep 7;9(9):986. doi: 10.3390/nu9090986 (PMC5622746; doi:10.3390/nu9090986)
Supplement: Supplementary file 1 [file nutrients-09-00986-s001.zip › Supplemental table S4.pdf]

**Supplemental Table S4.** Comparison of daily intake in micronutrients between quintiles (Q) of western pattern (N=44,350)

| | Q1 | | Q2 | | Q3 | | Q4 | | Q5 | | |
|---|---|---|---|---|---|---|---|---|---|---|---|
| | mean | SD | mean | SD | mean | SD | mean | SD | mean | SD | p |
| Calcium | 1025.2 | 2.86 | 917.7 | 2.80 | 877.0 | 2.77 | 853.0 | 2.80 | 809.2 | 3.05 | <0.0001 |
| Beta caroten | 4350.6 | 29.37 | 3533.2 | 28.79 | 3168.2 | 28.47 | 2906.5 | 28.76 | 2380.7 | 31.33 | <0.0001 |
| Iron | 14.1 | 0.05 | 13.5 | 0.05 | 13.1 | 0.05 | 12.9 | 0.05 | 12.1 | 0.05 | <0.0001 |
| Potassium | 3441.9 | 6.75 | 3025.3 | 6.61 | 2845.2 | 6.54 | 2716.2 | 6.61 | 2518.9 | 7.20 | <0.0001 |
| Magnesium | 371.6 | 1.20 | 344.1 | 1.17 | 332.6 | 1.16 | 322.1 | 1.17 | 301.6 | 1.28 | <0.0001 |
| Sodium | 2971.1 | 9.46 | 2943.4 | 9.27 | 2918.9 | 9.17 | 2871.1 | 9.26 | 2809.8 | 10.09 | <0.0001 |
| Phosphorus | 1408.2 | 3.14 | 1279.2 | 3.08 | 1227.4 | 3.05 | 1187.7 | 3.08 | 1132.3 | 3.35 | <0.0001 |
| Vitamin A | 1201.7 | 9.72 | 1076.8 | 9.53 | 1020.7 | 9.42 | 995.2 | 9.52 | 908.4 | 10.37 | <0.0001 |
| Vitamin B1 | 1.3 | 0.01 | 1.2 | 0.01 | 1.1 | 0.01 | 1.1 | 0.01 | 1.1 | 0.01 | <0.0001 |
| Vitamin B2 | 2.0 | 0.01 | 1.7 | 0.01 | 1.6 | 0.01 | 1.6 | 0.01 | 1.5 | 0.01 | <0.0001 |
| Vitamin B3 | 20.5 | 0.06 | 18.8 | 0.06 | 18.2 | 0.06 | 17.7 | 0.06 | 16.9 | 0.07 | <0.0001 |
| Vitamin B5 | 5.9 | 0.01 | 5.3 | 0.01 | 5.0 | 0.01 | 4.9 | 0.01 | 4.6 | 0.01 | <0.0001 |
| Vitamin B6 | 2.0 | 0.01 | 1.8 | 0.01 | 1.7 | 0.01 | 1.6 | 0.01 | 1.5 | 0.01 | <0.0001 |
| Vitamin B9 | 376.5 | 1.09 | 326.9 | 1.07 | 307.9 | 1.06 | 292.3 | 1.07 | 270.2 | 1.16 | <0.0001 |
| Vitamin B12 | 5.9 | 0.06 | 5.4 | 0.06 | 5.2 | 0.06 | 5.0 | 0.06 | 4.7 | 0.06 | <0.0001 |
| Vitamin C | 133.1 | 0.75 | 113.7 | 0.74 | 105.9 | 0.73 | 101.0 | 0.74 | 96.1 | 0.80 | <0.0001 |
| Vitamin D | 3.0 | 0.03 | 2.8 | 0.03 | 2.7 | 0.03 | 2.6 | 0.03 | 2.4 | 0.03 | <0.0001 |
| Vitamin E | 12.1 | 0.05 | 11.3 | 0.05 | 10.9 | 0.04 | 10.6 | 0.05 | 10.1 | 0.05 | <0.0001 |
| Zinc | 11.5 | 0.03 | 10.9 | 0.03 | 10.5 | 0.03 | 10.2 | 0.03 | 9.6 | 0.03 | <0.0001 |

ANCOVA tests controlled for gender, age and total energy intake

Abbreviations: SD standard deviation
